# Enzyme activity and structural features of three single-domain phloem cyclophilins from *Brassica napus*

**DOI:** 10.1038/s41598-019-45856-y

**Published:** 2019-06-27

**Authors:** Patrizia Hanhart, Sven Falke, Marcel Garbe, Victoria Rose, Melanie Thieß, Christian Betzel, Julia Kehr

**Affiliations:** 1 Universität Hamburg, Institute of Plant Science and Microbiology, Molecular Plant Genetics, Ohnhorststraße 18, 22609 Hamburg, Germany; 2 Universität Hamburg, Institute of Biochemistry and Molecular Biology, Laboratory for Structural Biology of Infection and Inflammation, c/o DESY, Notkestraße 85, 22603 Hamburg, Germany

**Keywords:** Enzymes, SAXS, X-ray crystallography, Plant sciences

## Abstract

Cyclophilins (CYPs) are a group of ubiquitous prolyl *cis*/*trans* isomerases (PPIases). It was shown that plants possess the most diverse CYP families and that these are abundant in the phloem long-distance translocation stream. Since phloem exudate showed PPIase activity, three single-domain CYPs that occur in phloem samples from *Brassica napus* were characterised on functional and structural levels. It could be shown that they exhibit isomerase activity and that this activity is controlled by a redox regulation mechanism, which has been postulated for divergent CYPs. The structure determination by small-angle X-ray scattering experiments revealed a conserved globular shape. In addition, the high-resolution crystal structure of BnCYP19-1 was resolved and refined to 2.0 Å resolution, and the active sites of related CYPs as well as substrate binding were modelled. The obtained data and results support the hypothesis that single domain phloem CYPs are active phloem PPIases that may function as chaperones.

## Introduction

Cyclophilins (CYPs) are ubiquitous proteins involved in a number of fundamental cellular functions in a large number of organisms, such as animals, plants, fungi, bacteria and viruses^1^. Together with the structurally unrelated FK506-binding proteins (FKBPs), they belong to the superfamily of immunophilins that have originally been discovered as proteins binding to the immunosuppressant peptide drug cyclosporin A (CsA)^2^ or FK506/rapamycin^3^, respectively. Many proteins of both groups possess a peptidyl-prolyl *cis/trans* isomerase (PPIase) activity, implemented by the conserved FKBP- or CYP-like domain (CLD). By these isomerases the transition from *cis* to *trans* in an X-proline peptide bond, a rate-limiting step in protein folding^4,5^, is stabilised or accelerated. Furthermore, CYPs may also be involved in signalling^6^, pathogen response^7^, RNA processing^8,9^ gene repression^10^, as well as plant stress responses and development^11,12^.

Interestingly, plants possess the most diverse CYP families with rice (*Oryza sativa*) encoding 27^13^, *Arabidopsis thaliana* encoding 29^14^, soybean (*Glycine max*) encoding 62^15^, and oilseed rape (*Brassica napus*) encoding 91^16^ distinct CYP proteins. *A*. *thaliana* and *B*. *napus*, both belonging to the family of *Brassicaceae*, are of great importance as model organisms and in the case of *B*. *napus* also in agriculture. As has already been shown for various plant species, CYPs are abundant proteins in the phloem long-distance transport stream and it is assumed that they support protein refolding after trafficking into sieve elements^17–21^. With only few exceptions, functions of phloem CYPs are so far unknown. CYP1 from tomato (SlCYP1), however, has been suggested to be involved in long-distance signalling modulating auxin responses^22^.

Twenty distinct CYPs have been identified in the phloem of *B*. *napus* and all of them belong to the family of single-domain CYPs^16^. They are composed of the CLD with a common structure motif of an eight anti-parallel stranded right-handed β-barrel with two α-helices at the top and bottom^23^. Investigation of the most widely studied CYP, human CYPA (also known as hCYPA or HsCYPA), led to the identification of its CsA binding site^24^. Since the first structure of HsCYPA has been determined, four CYP structures from plants have been resolved (summarised in^25^). In contrast to the investigated CYPs from *Citrus sinensis* (CsCYP)^26^, *Triticum aestivum* (TaCYPA-1)^27^, and *Catharanthus roseus* (Cat r 1)^28^, which all constitute single-domain variants, *A*. *thaliana* AtCYP38 is a multi-domain protein consisting of the CLD plus a PsbQ-like helical bundle^29^. Yet, none of these proteins was assigned to the phloem. Since the structure of the tomato phloem CYP SlCYP1 has only been modelled^22^, experimental validation of a phloem mobile CYP structure is still missing.

The identification of CYPs in the phloem of *B*. *napus* under standard growth conditions supports the assumption that these proteins fulfil essential functions and may act as chaperones. In this context, the first question arising is whether CYPs can exercise their isomerase activity in the phloem. Therefore, we studied not only the PPIase activity of *B*. *napus* phloem exudate, but also of individual CYPs. The investigated candidate proteins BnCYP18-4, BnCYP18-5, and BnCYP19-1 were chosen because of their homology to already examined plant CYPs, either known to be phloem localised or from the close relative *A*. *thaliana*. Concerning AtCYPs, we included AtCYP19-3 into the study, which has not only been previously analysed in activity assays and by *in silico* modelling^30^, but also resembles the closest *A*. *thaliana* homolog to one of the investigated *B*. *napus* CYPs, BnCYP19-1. Small-angle X-ray scattering (SAXS) experiments of all four selected CYPs were performed to verify and compare their overall structure in solution. In addition, the high resolution structure of one phloem CYP, BnCYP19-1, was determined by X-ray crystallography. These data were further utilised to model active site residues of the other CYPs. The results show that the small specific activity differences observed cannot be explained by the conformation of the catalytic and CsA-binding residues alone.

## Results and Discussion

### *B*. *napus* phloem exudate has peptidyl-prolyl *cis*/*trans* isomerase activity

**Figure 1:**
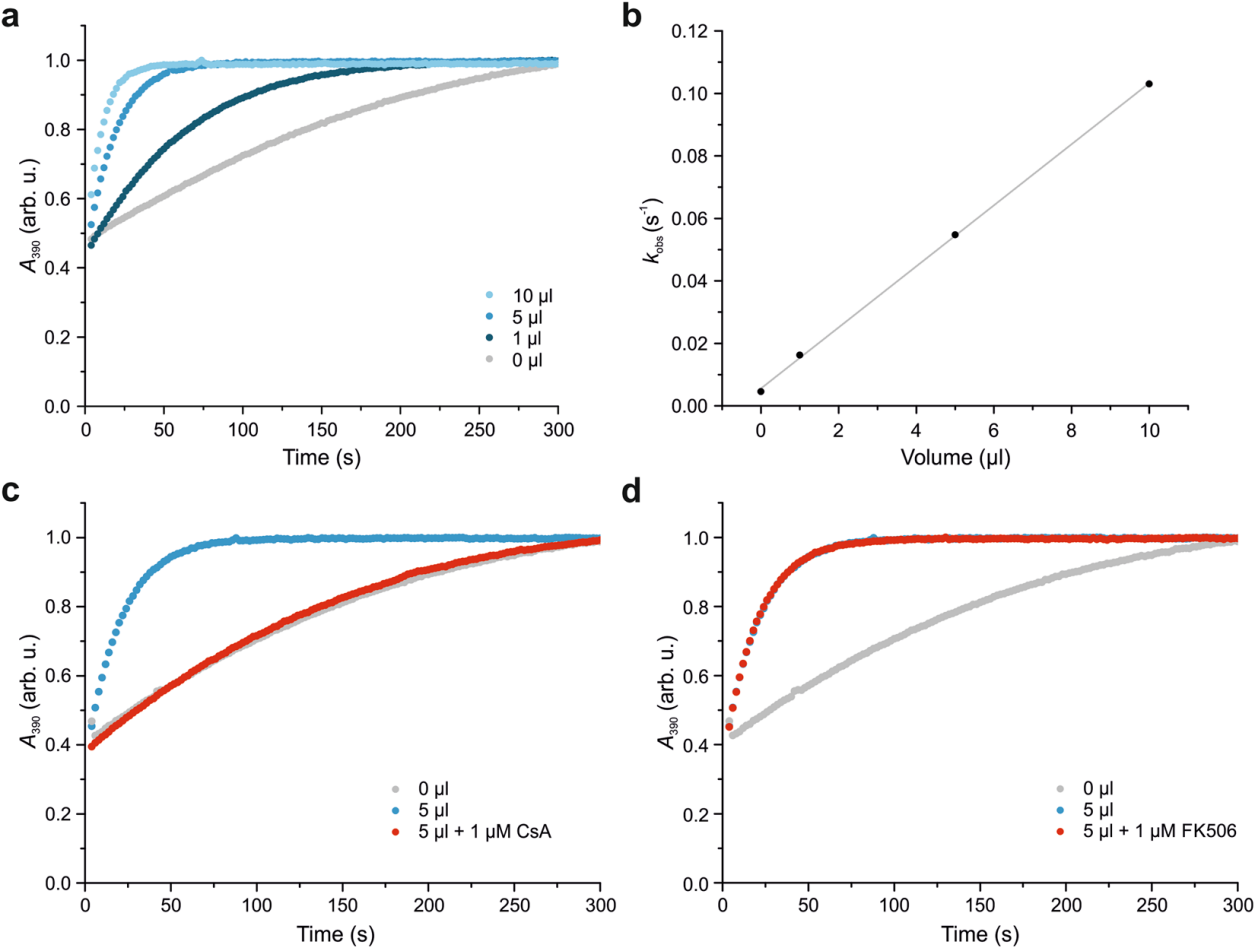
*B*. *napus* phloem exudate has peptidyl-prolyl *cis*/*trans* isomerase activity. (**a**) Increasing amounts of phloem exudate show increasing catalytic activity. (**b**) The rate constants *k*_obs_ demonstrate a linear behaviour. (**c**) The activity can be reduced by the addition of the cyclophilin inhibitor CsA. (**d**) The FKBP inhibitor FK506 does not influence the isomerisation rate.

To support the hypothesis of CYPs being active PPIases in the phloem, the activity of freshly sampled phloem exudate was measured. A common assay to assess the isomerisation rate of PPIases has been first described by Fischer *et al*. in^31^. Mostly purified or recombinantly expressed proteins were investigated by this method, but it has also been applied to protein mixtures. In an attempt to answer the question whether PPIases are active in phloem exudate of *Brassicaceae*, we sampled *B*. *napus* phloem sap and added it directly to the assay mixture, what resulted in an enhanced isomerisation reaction (Fig. 1a). The observed rate constants showed a linear increase correlated with increasing amounts of phloem exudate (Fig. 1b). It is assumed that this activity results from a mixture of active CYPs, since 20 distinct CYPs have been identified in the phloem^16^. After the addition of CsA, a well-known cyclophilin inhibitor, the activity was reduced (Fig. 1c). In contrast, the addition of FK506, a FKBP inhibitor, did not result in any activity changes (Fig. 1d), demonstrating that the activity originates only from CYPs. Similar observations have been described for phloem exudate from *Ricinus communis*, a member of the family of *Euphorbiaceae*^32^.

### Sequence similarities of plant single-domain CYPs

A challenge in studying highly similar proteins is to provide evidence that small differences in their sequence affect activities or structural arrangements. The three closely related BnCYPs examined in this study, BnCYP18-4, BnCYP18-5, and BnCYP19-1, are showing only minor variances in their amino acid composition (Supplementary Fig. S1), resulting in high sequence identities of 74 to 82%. In comparison with single-domain CYPs from other plant species, such as the *A*. *thaliana* homolog AtCYP19-3, the structurally resolved citrus CsCYP^26^ and wheat TaCYPA-1^26,27^, the phloem CYPs SlCYP1 from tomato^22^, and RcCYP1 from castor bean^32^, the high sequence conservation even between different species becomes obvious. The closest *B*. *napus* homolog of SlCYP1 is BnCYP18-5 with a sequence identity of 83%, and BnCYP18-4 has a sequence identity of 85% with RcCYP1. To compare CYPs from the family of *Brassicaceae*, AtCYP19-3 has been chosen which shares 91% sequence identity with BnCYP19-1.

All of the amino acid residues that have been experimentally demonstrated to be important for PPIase activity, CsA binding or disulphide bridge formation^26,33,34^ are conserved in all comparatively analysed CYPs, except for Cys40, which is missing in HsCYPA. Furthermore, in contrast to HsCYPA all surveyed plant CYPs show an insertion which occurs in many plant^14^, nematode^35^, and human^36^ CYPs and is located within amino acid region 48 to 54 (Supplementary Fig. S1). These amino acids are positioned between α-helix-I and β-sheet-III (the so-called α-I/β-III junction^14^ or divergent loop^37^) and result in an extended loop region.

### Activity of individual phloem CYPs

In order to find out whether the most abundant BnCYPs in the phloem, the 18–19 kDa representatives, contribute to its PPIase activity, we directly compared the catalytic activities of three abundant phloem single-domain CYPs from *B*. *napus*, BnCYP18-4 (18.6 kDa), BnCYP18-5 (18.7 kDa), BnCYP19-1 (19.9 kDa), and the *A*. *thaliana* AtCYP19-3 (19.2 kDa). The four proteins were expressed in *E*. *coli* and purified to homogeneity after cleaving off the His-tag.

**Figure 2:**
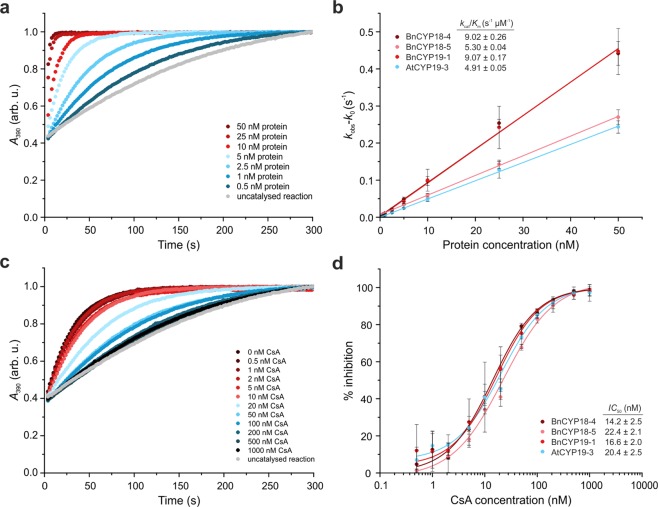
Determination of the enzymatic activity of recombinant *Brassica napus* CYPs and their inhibition by CsA. (**a**) Representative normalised raw data of BnCYP18-4 measured in a concentration range of 0.5 to 50 nM protein. (**b**) Influence of the protein concentration on the rate constant. (**c**) Representative normalised raw data of BnCYP18-4 inhibition by CsA with inhibitor concentrations of 0 to 1000 nM. (**d**) Dose-response curves of BnCYP18-4, BnCYP18-5, BnCYP19-1, and AtCYP19-3.

While the PPIase assay raw data confirmed the intuitive concept that the more enzyme present, the higher the activity (Figs 2a, S2), plotting *k*_obs_-*k*_0_ versus the protein concentration showed a linear growth of the rate constant of the catalysed isomerisation reaction (Fig. 2b). The results showed that the surveyed CYPs were active PPIases with catalytic efficiencies in a similar range as reported for other plant CYPs (summarised in^30^). BnCYP18-4 had a catalytic activity *k*_cat_/*K*_m_ of 9.02 ± 0.26 s^−1^ µM^−1^ and is therewith similarly active as BnCYP19-1 with 9.07 ± 0.17 s^−1^ µM^−1^. BnCYP18-5 had a slightly lower catalytic activity (5.30 ± 0.04 s^−1^ µM^−1^). The activity of AtCYP19-3 has been measured in a previous study with a *k*_cat_/*K*_m_ value of 2.7 s^−1^ µM^−1^ ^30^. In our study its activity was higher (4.91 ± 0.05 s^−1^ µM^−1^) what might be caused by optimisation of the assay set-up. For comparison, a catalytic activity of 1.4 s^−1^ µM^−1^ has been reported for HsCYPA^38^.

The addition of the inhibitor CsA resulted in reduced isomerisation rates (Fig. 2c) that can be visualised as a dose-response curve (Fig. 2d). All four CYPs bound CsA and concentrations above 500 nM CsA inhibited the reaction completely. The *IC*_50_ values of the inhibition kinetics showed that BnCYP18-4 (14.2 ± 2.5 nM), BnCYP18-5 (22.4 ± 2.1 nM), BnCYP19-1 (16.6 ± 2.0 nM), and AtCYP19-3 (20.4 ± 2.5 nM) bound CsA with only small variances in affinity.

### Redox regulation of enzyme activity

**Figure 3:**
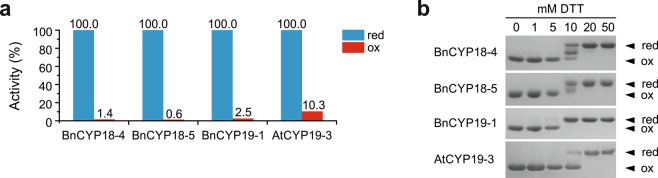
Redox regulation of PPIase activity via disulphide bond formation. (**a**) Reduced protein shows high activity in the PPIase assay, but its oxidation results in drastically reduced isomerisation rates. (**b**) Titrating reducing (0-50 mM DTT) versus oxidising (10 mM Cu^2+^) agent leads to an altered migration pattern on a non-reducing SDS-PAGE. Cropped gels are shown for clarity. Full size gels are presented in Supplementary Fig. S3. Oxidation by H_2_O_2_ showed similar results.

Since all investigated CYPs belong to the divergent CYPs which were earlier proposed to be controlled by a redox regulated mechanism^26^ they were additionally subjected to activity assays under oxidative and reducing conditions. While BnCYP18-4, BnCYP18-5, BnCYP19-1, and AtCYP19-3 were highly active after reduction, all enzymes lost their activity after oxidative treatment (Fig. 3a). This is suggested to result from the formation of a disulphide bridge between Cys40 and Cys168, altering the enzyme structure allosterically via the divergent loop^26^. Supporting this hypothesis, a titration experiment of reducing versus oxidising agent revealed a redox state dependent migration pattern (Fig. 3b). Such changes in the migration behaviour are suggested to result mainly from the formation of disulphide bridges as it could be shown earlier for AtCYP20-3^34^.

### In solution protein shape

Matching the PPIase assay data, the same four CYPs, BnCYP18-4, BnCYP18-5, BnCYP19-1, and AtCYP19-3 were structurally investigated by SAXS experiments. Beforehand, the samples were analysed by dynamic light scattering (DLS). This demonstrated that all four proteins were monomeric in solution (Supplementary Fig. S4). The hydrodynamic radii (*R*_h_) are provided in Table 1. After this quality assessment monodisperse sample solutions were subjected to SAXS in order to compare the radii of gyration (*R*_g_) and maximum diameters (*D*_max_) (Table 1), as well as to model the tertiary structure.

**Table 1:** Size and *ab initio* model parameters of single-domain plant CYPs according to SAXS and DLS experiments, respectively.

	BnCYP18-4	BnCYP18-5	BnCYP19-1	AtCYP19-3
**Sample description**				
Gene ID	*BnaC03g60160D*	*BnaA09g08780D*	*BnaA09g35540D*	*At3g56070*
UniProt ID	A0A078DMP0	A0A078IDN6	A0A078GRH6	Q28867
Extinction coefficient (*A*_280_, M^−1^ cm^−1^)	8480	11460	9970	9970
*MW*_theor_ (kDa)	18.6	18.7	19.9	19.2
**Structural parameters**				
**DLS**				
*R*_h_ (nm)	2.1 ± 0.1	2.4 ± 0.4	2.3 ± 0.1	3.0 ± 0.2
**Guinier analysis**				
*I*(0)	0.027 ± 0.00005	0.028 ± 0.000054	0.031 ± 0.000039	0.031 ± 0.000037
*R*_g_ (nm)	1.58 ± 0.22	1.60 ± 0.06	1.72 ± 0.02	1.70 ± 0.22
*s*_min_ (nm^−1^)	0.12	0.16	0.17	0.15
*sR*_g_ max	1.30	1.30	1.30	1.30
*R* ^2^	0.90	0.94	0.96	0.96
^*^ *MW*_*I*(0)_	14.9	15.4	17.1	17.1
***P*** **(** ***r*** **) analysis**				
*I*(0)	0.030 ± 0.00007	0.023 ± 0.00004	0.027 ± 0.00004	0.028 ± 0.00003
*R*_g_ (nm)	1.56 ± 0.005	1.57 ± 0.003	1.72 ± 0.003	1.68 ± 0.002
*D*_max_ (nm)	5.3	4.6	5.9	5.7
Porod Volume *V*_p_ (nm^−3^)	28.36	33.12	29.00	33.12
**Modelling**				
**DAMMIF**				
Symmetry, anisotropy	P1, unknown	P1, unknown	P1, unknown	P1, unknown
*χ* ^2^	1.408	1.239	1.297	1.606
mean NSD	0.887 ± 0.037	1.009 ± 0.053	1.084 ± 0.100	0.928 ± 0.048
Crystal structure for homology modelling	CsCYP with CsA	CsCYP with CsA	CsCYP with CsA	CsCYP with CsA
PDB ID	4JJM	4JJM	4JJM	4JJM
**CRYSOL**				
*χ* ^2^	1.051	1.176	3.061	2.460

*Estimated from the forward scattering intensity, according to *I*(0) ~ *MW*.

**Figure 4:**
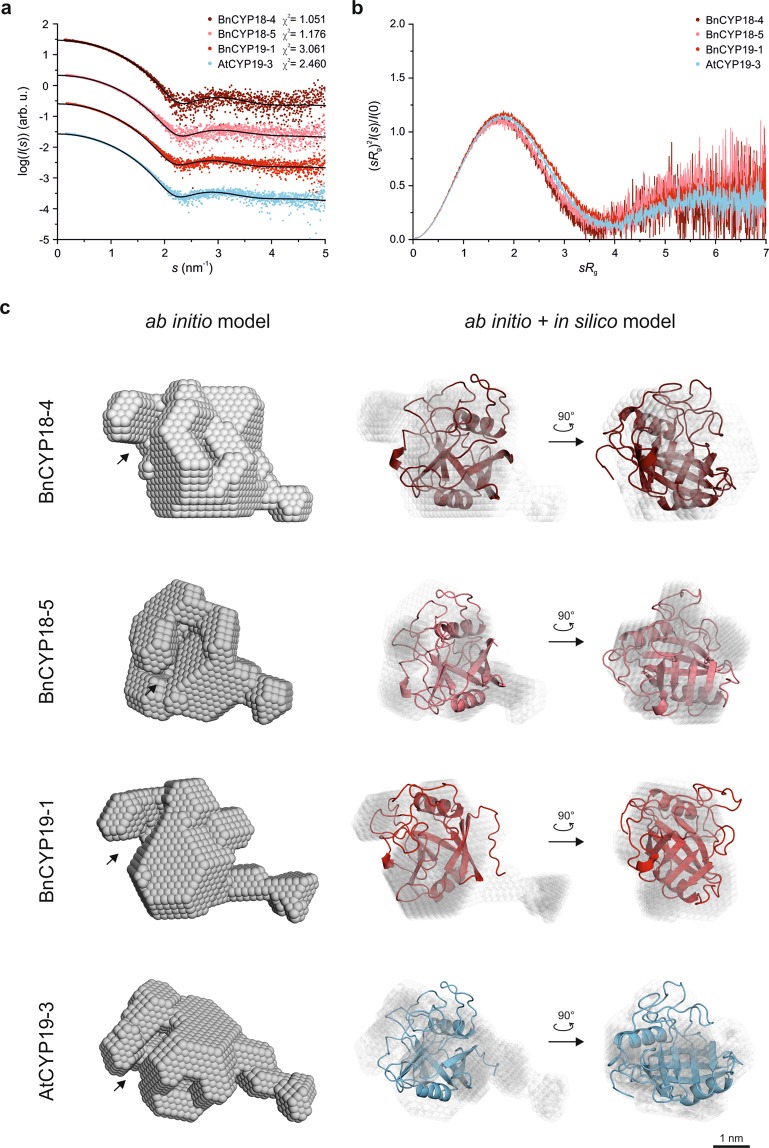
Comparative SAXS data and solution structures. (**a**) X-ray scattering intensities depending on the momentum transfer *s* are shown. In addition, the theoretical scattering pattern calculated by CRYSOL based on the respective *in silico* model (depicted in c) is shown as well. The intensity distributions are displaced vertically for clarity. (**b**) Dimensionless Kratky plot. (**c**) Representation of a single *ab initio* shape of each CYP and its comparison to the *in silico* models. The putative substrate binding sites are indicated by black arrows.

The SAXS data (Fig. 4a) strongly indicated a conserved nearly globular shape, which was also predicted by *ab initio* modelling (Fig. 4c). The CYP domain is consisting of a compact globular structure with an “opened” cleft, resembling the binding site of substrates and ligands like CsA. The dimensionless Kratky plots of all four CYPs are similar with a slight shift along the vertical axis for BnCYP19-1 and AtCYP19-3 at higher scattering angles (Fig. 4b). This observation might be related to elongated, flexible termini of the slightly larger CYPs. Supporting this observation, the *ab initio* models also strongly indicate a flexible C-terminus for all four CYPs. This flexibility is in agreement with the higher *χ*^2^ value of the BnCYP19-1 and AtCYP19-3 CRYSOL fit curves compared to these of BnCYP18-4 and BnCYP18-5. The addition of CsA resulted in no significant changes of the overall three-dimensional shape of the CYPs (data not shown), which is consistent with previous results for HsCYPA^39^.

### High-resolution structure of a phloem CYP

**Figure 5:**
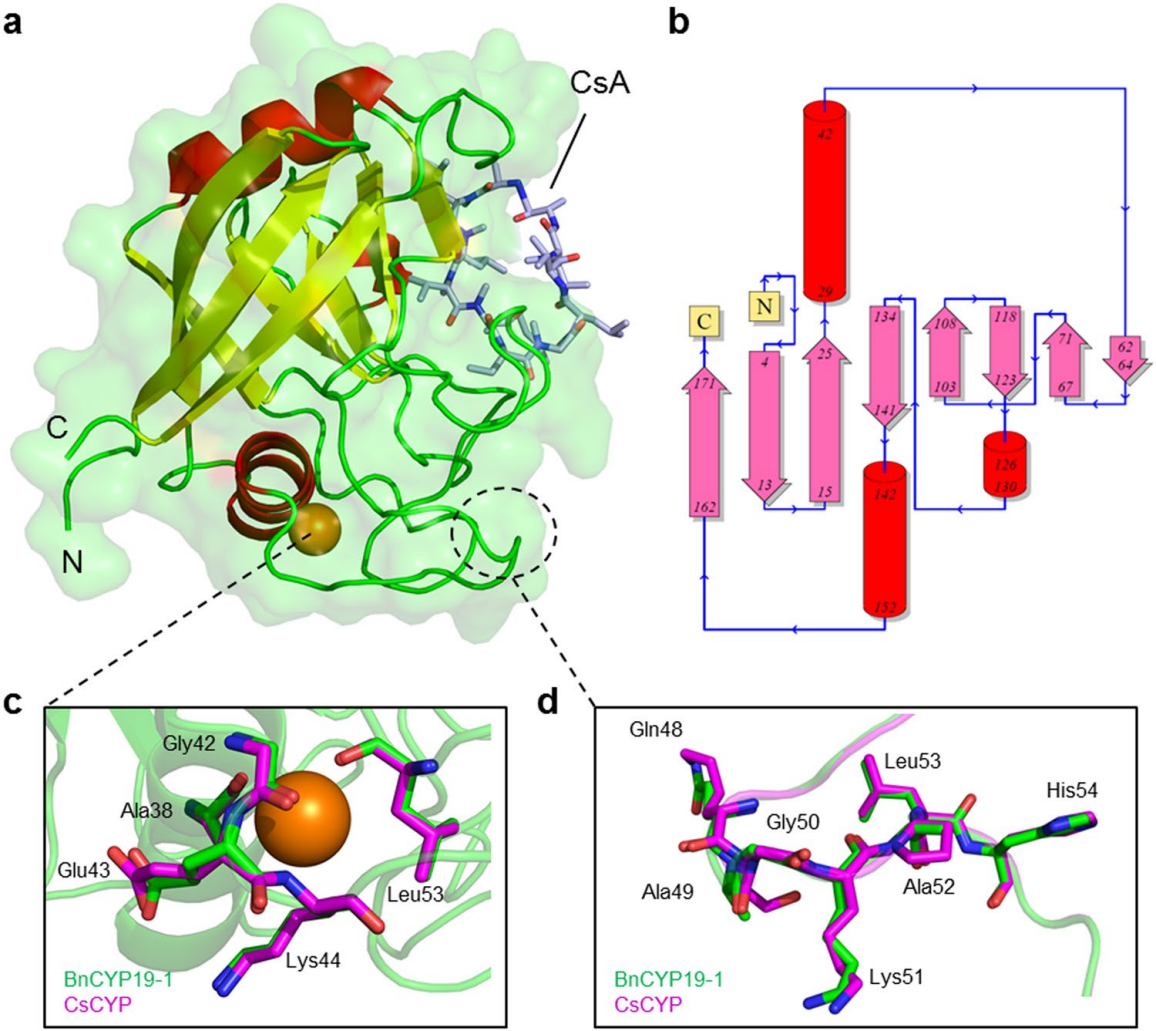
Crystal structure of BnCYP19-1. (**a**) Cartoon plot of BnCYP19-1. (**b**) Topology of the BnCYP19-1 secondary and tertiary structure as visualised by pdbsum (EMBL-EBI^65^). (**c**) Cation binding site of BnCYP19-1 superimposed with CsCYP. (**d**) Superimposition of the divergent loop residues 48-54. BnCYP19-1 is coloured green and the homologous loop of CsCYP in magenta for comparison. A cross-eye stereo image of a section of the BnCYP19-1 electron density map with a resolution of 1.98 Å is provided in Supplementary Fig. S6.

After verifying the monomeric state and overall protein shape of selected CYPs in solution, the crystal structure of BnCYP19-1 in complex with CsA (Fig. 5a,b) was resolved with one molecule per asymmetric unit and refined to a resolution of 2.0 Å. The tertiary structure is overall conserved compared to the plant homologues CsCYP and TaCYPA-1 (pdb id: 4JJM and 4E1Q), which share 67% and 66% sequence identity with BnCYP19-1, respectively. Also the homology models of BnCYP18-4, BnCYP18-5 and AtCYP19-3, which were calculated *in silico* and are based on the atom coordinates of BnCYP19-1, indicate a high degree of structural conservation compared to each other. The calculated respective RMS values for main chain atoms are around 1 Å, and the overall fold is highly similar compared to HsCYPA as well. BnCYP19-1 possesses a conserved β-barrel-like structure, consisting of eight β-sheets interconnected by three α-helices. Interestingly, the structure of BnCYP19-1 has a previously undescribed site for coordinative metal binding being occupied by Mg^2+^ (Fig. 5c). However, Mg^2+^ did not show any influence on the enzyme activity. Furthermore, the crystal structure revealed a malonate molecule forming non-bonded hydrophobic contacts with Ile46 and Gly47. This binding site might interact with an unknown small organic metabolite *in vivo*. While discussing the crystal structure it should also be noted that the C-terminus of BnCYP19-1 is not well-defined by the electron density map. BnCYP19-1 possesses additional disordered amino acids that are absent in other CYPs (Supplementary Fig. S1).

### Structure and regulation of the active site

**Figure 6:**
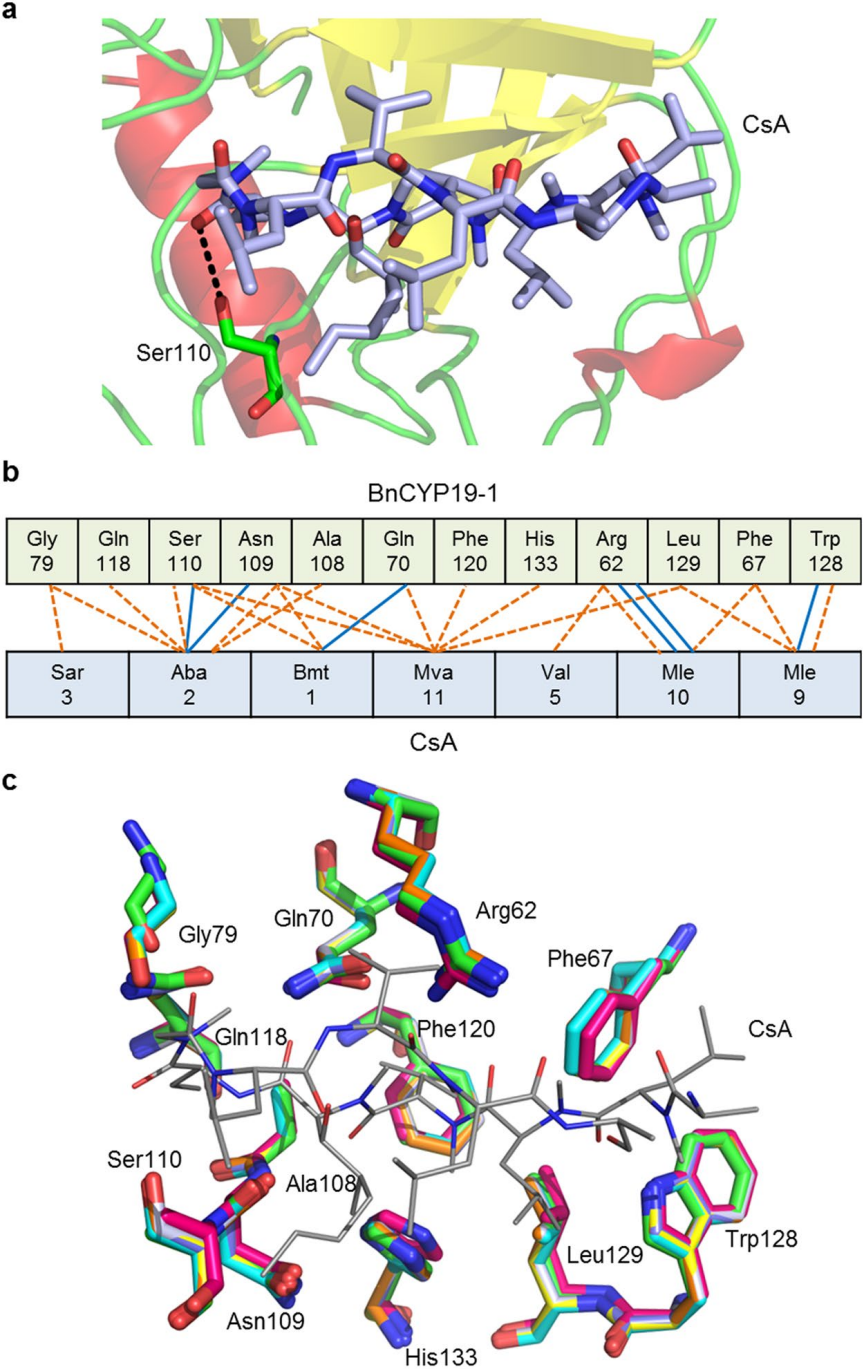
Active site structure. (**a**) Active site of BnCYP19-1 with bound CsA. Serine 110 is highlighted. (**b**) Schematic representation of the BnCYP19-1 binding epitope according to pdbsum (EMBL-EBI^65^) with hydrogen bonds indicated by blue lines and hydrophobic interaction indicated by orange dashed lines. (**c**) Superimposition of active site residues involved in substrate and CsA interaction (crystal structure of BnCYP19-1 in cyan, BnCYP18-4 in purple, AtCYP19-3 in orange, BnCYP18-5 in beige, CsCYP in green and native TaCypA-1 without CsA (pdb ID: 4E1Q) in magenta). The active site geometry is widely conserved also in TaCYPA-1 in the absence of an active site compound.

Most of the residues involved in substrate recognition and CsA binding are conserved with similar orientations (Fig. 6b,c). Therefore, it is consistent that the observed enzymatic activities and CsA binding kinetics are in a similar range for all CYPs examined.

Despite this fact, there are several structural differences. In contrast to most CYPs, including the two plant homologues from *C*. *sinensis* and *T*. *aestivum*, both BnCYP19-1 and AtCYP19-3 possess a serine at position 110 instead of an alanine. The high-resolution structure of BnCYP19-1 showed that the additional hydroxyl group is oriented towards a carbonyl oxygen (Aba2-O) of CsA, providing an additional hydrogen bond (2.9 Å, Fig. 6a). Thereby, binding to CsA is stabilised by a total of 6 hydrogen bonds involving 5 amino acids of BnCYP19-1 (Supplementary Table S3). Still, this additional bond does not result in an increased binding capacity.

In case of substrate binding, modelling approaches via superimposition based on the epitope of a Gly-Pro dipeptide binding to HsCYPA^40^ and an oligopeptide binding to a bacterial CYP^41^, showed that Ser110 may not interact with the proline peptide bond that is catalytically isomerised. But it could still alter the core substrate specificity.

In order to identify the binding epitope of the activity assay peptide substrate, Suc-AAPF-pNA, at its BnCYP19-1 binding site, *in silico* docking was performed (Supplementary Fig. S5). Data obtained suggest a hydrogen bond via the hydroxyl group of Ser110 with the succinyl moiety and a hydrogen bond of Arg62 with the backbone carbonyl oxygen of the phenylalanine residue of the substrate among others within the elongated binding cleft. Given the conservation of many active site residues, the differences observed in catalytic activity of the substrate are rather explainable by non-conserved long range interactions and turnover limiting conformational changes of the whole protein as described for HsCYPA^42,43^ involving particularly residues 75–85, 101–110 and 147–155, which correspond to 82–92, 108–117 and 154–162 in BnCYP19-1. Further the relatively high B-values of Arg70, Lys154 and Arg159 of BnCYP19-1, which are not fully conserved, indicate an involvement in such a mechanism. Previous studies of HsCYPA applied NMR spectroscopy and MD simulations to probe tertiary structure conformational changes, which could further be studied in BnCYP19-1 using time-resolved X-ray crystallography similar to XFEL experiments performed with HsCYPA^44^.

Discussing the redox regulation model from Campos *et al*.^26^ in the context of our results from the activity assays and the structural analysis, it becomes obvious that the divergent BnCYPs and AtCYPs may be regulated by the same mechanism. For CsCYP it is reported that the formation of a disulphide bond between Cys40 and Cys168 induces a repositioning of α-helix I. Thereby hydrogen bonds of Glu83 with Lys48 and Ser49 are disrupted. In BnCYP19-1 (Fig. 5d), Glu83 forms hydrogen bonds with the backbone amide nitrogen of Gln48 (2.8 Å) and Ala49 (3.4 Å), respectively. Due to the alanine in position 49, the hydrogen bond of the Ser49 side chain reported for CsCYP cannot be formed. This leads to only two hydrogen bonds connecting the divergent loop to Glu83. The activity assays under oxidative treatment showed that this is still sufficient to provide the redox regulation mechanism via this interaction. According to the X-ray crystal structure of BnCYP19-1, all four Cys residues were clearly in a reduced state.

## Conclusions

Specific CYPs are highly abundant in the phloem of *B*. *napus* and other plant species. In phloem exudate of *Brassica napus*, 18 and 19 kDa single-domain CYPs have been detected in 8 distinct spots on 2D-PAGE gels^17^ and 8 were detected by LC-MS/MS, with BnCYP18-5 being by far the most abundant CYP^16^.

In the present study we have compared three similar *B*. *napus* phloem CYPs plus a homologous *A*. *thaliana* CYP by enzyme activity assays and by structural analysis to understand if the minor differences in amino acid sequences alter structural or functional features.

The expressed and purified three phloem CYPs all showed PPIase activity with only minor differences in activity and inhibitor affinity. Furthermore, they may be regulated by a redox controlled mechanism. A crystal structure at 2 Å resolution enabled the analysis of the inhibitor binding cleft and suggested together with substrate docking no significant changes at the catalytic binding site, except for the non-conserved amino acid position 110.

In summary, our data confirm that the single domain phloem CYPs function as phloem PPIases and as protein chaperones, as it has been proposed earlier^17,32^. Because of the high number of CYPs in the phloem of *B*. *napus* and their high sequence similarity as well as functional and structural redundancy, it seems unlikely that these CYPs, in contrast to SlCYP1 in tomato^22^, function as long-distance signalling molecules. The data rather lead to the conclusion that the necessity of multiple enzymes may either result from their fundamental role which has to be maintained even under loss of individual CYP isoforms or from the modulation of substrate specificity by residues surrounding the catalytic cleft.

## Methods

### Multiple sequence alignment of CYP proteins

Sequences were aligned using ClustalOmega (http://www.ebi.ac.uk/Tools/msa/clustalo/)^45^ and displayed with Jalview 2.9.0b2^46^. For comparison, RcCYP1 (UniProt KB ID: Q8VX73), SlCYP1 (P21568), HsCYPA (P62937), CsCYP (D0ELH5), and TaCYPA-1 (Q93W25) were aligned together with the examined oilseed rape and Arabidopsis cyclophilins.

### Plant material, growth conditions and phloem exudate collection

*Brassica napus* cultivar ‘Drakkar’ plants were grown in 19 cm pots on soil (LAT-Terra Standard P, Industrie-Erdenwerk Archut, Germany) under controlled conditions in a glasshouse. The conditions applied were 70% humidity and a 16 h/8 h light/dark (day/night) with 22 °C/18 °C (day/night) cycle. Plants were watered once per day and fertilized with 2 g l^−1^ Osmocote Exact Standard High K (Scotts, the Netherlands). Phloem exudate was collected by exudation as described by Giavalisco *et al*.^17^ and used for the determination of its PPIase activity directly after collection.

### Cloning, gene overexpression and purification

The open reading frames encoding BnCYP18-4 (*BnaC03g60160D*), BnCYP18-5 (*BnaA09g08780D*), BnCYP19-1 (*BnaA09g35540D*), and AtCYP19-3 (*At3g56070*) were amplified by PCR from genomic DNA of either *Brassica napus* cultivar ‘Drakkar’ or *Arabidopsis thaliana* ecotype Col-0 and cloned to pET28a+ with NdeI/XhoI (primers are listed in Supplementary Table S1).

*Escherichia coli* BL21 (DE3) were transformed with the pET28a+ constructs in order to produce His_6_-tagged BnCYP18-4, BnCYP18-5, BnCYP19-1, and AtCYP19-3 via recombinant gene overexpression. Liquid cultures in 2YT medium containing kanamycin (50 µg ml^−1^) were incubated at 37 °C and supplemented with IPTG at a final concentration of 1 mM after the optical density at 600 nm reached 1.0 AU. After 3 hours induction the cells were harvested by centrifugation and resuspended in lysis buffer (20 mM Tris/HCl pH 7.4, 300 mM NaCl, 2 mM imidazole) for cell disruption by lysozyme (f. c. 1 mg ml^−1^) and sonication. After centrifugation at 43000 x g the clarified supernatant was applied to Ni-NTA affinity chromatography and the His_6_-tagged proteins were eluted with 20 mM Tris/HCl pH 7.4, 300 mM NaCl, 200 mM imidazole. To remove imidazole and cleave the tag, thrombin digestion was combined with dialysis in 20 mM Tris/HCl pH 7.4, 300 mM NaCl. Subsequently, further purification was achieved by size exclusion chromatography. Finally, the proteins were either concentrated to the desired protein concentration and directly used or they were concentrated to 10 mg ml^−1^, supplied with 5% (v/v) glycerol and 0.1 mM AeBSF and stored at −80 °C. In order to check for purity, SDS-PAGE and MALDI-TOF mass spectrometry were carried out.

### Non-reducing SDS-PAGE to visualise disulphide bond formation

Protein (25 µM) was pre-treated with oxidising agent (CuSO_4_, 10 mM) for 15 min at room temperature. The mixture was split and each aliquot was mixed with varying amounts of DTT (1-50 mM). After adding SDS sample buffer without reducing agent (60 mM Tris/HCl pH 6.8, 2% SDS, 10% glycerol, 0.02% bromophenol blue) 1 µg protein was directly loaded onto a 15% SDS-PAGE and stained with colloidal coomassie^47^.

### Comparative determination of PPIase activity

Based on the protease-coupled PPIase assay described by Fischer *et al*.^31^, the PPIase activity was determined in 35 mM HEPES pH 8.0 as assay buffer and the whole approach was carried out at 4 °C. Either phloem exudate (1, 5 or 10 µl) or purified protein was added. Purified proteins were pre-diluted in assay buffer to stock solutions of 2.5 µM and 250 nM. From these different protein concentrations (0.5 to 50 nM) were prepared with assay buffer and pre-incubated for 5 min. 12 µM α-Chymotrypsin (from a stock solution of 2.4 mM in 1 mM HCl) as well as 80 µM substrate (from a stock solution of 8 mM) were added directly before the measurement. As a substrate N-Succinyl-Ala-Ala-Pro-Phe-p-Nitroanilide (short Suc-AAPF-pNA) was solubilised in 470 mM LiCl, 100% 2,2,2-Trifluorethanol (as described by Kofron *et al*.)^48^. After the addition, the total volume of 1 ml was mixed for 4 s and the absorption at 390 nm was recorded with 2 s intervals for 300 s. Three replicates were recorded for each protein concentration. The data were fitted with the first order reaction equation *y* = *y*_0_ + *a* e^−*kt*^ (with *k* as the observed rate constant *k*_obs_). The rate constant of the isomerase was determined by subtracting *k*_0_ from *k*_obs_ (with *k*_0_ as the spontaneous uncatalysed *cis/trans* isomerisation rate) and these values were plotted against the protein concentration. The data points could be fitted with a linear regression where the slope is *k*_cat_/*K*_m_.

To determine the activity after oxidative or reductive treatment, 25 µM protein was pre-treated with either 10 mM CuSO_4_ or 100 mM DTT for 15 min at room temperature and applied to the assay mixture as described above.

### Inhibition of PPIase activity

To determine the inhibitory potential of CsA the PPIase assay was performed as described above except that the protein concentration was kept constant at 5 nM while the CsA concentration ranged from 0 to 1000 nM. To examine the inhibition of phloem exudate activity 10 µl phloem sap was applied and either 1000 nM CsA or FK506 were added.

CsA was solubilised in 100% DMSO to 10 mM and diluted to appropriate stock solutions with the same solvent. After adding the appropriate amount of CsA to the assay mixture, pure DMSO was added if necessary to keep the total amount of solvent constant at 0.1%. The assay mixture was incubated for 5 min at 4 °C before the reaction was started. The PPIase activity without any CsA was defined as maximal active (=*k*_max_) so that the inhibition could be calculated by *y* = (*k*_max_ − (*k*_obs_-*k*_0_))/*k*_max_. The obtained values were plotted against the CsA concentration. Changing the x-axis to a logarithmic scale resulted in a sigmoid shape of the data points which could be fitted with *y* = *A*_1_ + (*A*_2_ − *A*_1_)/(1 + (*x*/*x*_0_)^*p*^) to determine the *IC*_50_.

### Dynamic light scattering (DLS)

Solution dispersity and oligomeric states of CYPs were determined using DLS. Protein solution aliquots in a quartz cuvette were exposed to a 660 nm laser applying a Spectrolight 300 instrument (Xtal Concepts, Germany). The time-dependent fluctuations of the scattering intensity at an angle of 90° were autocorrelated and evaluated using the CONTIN algorithm^49^. The determined diffusion coefficients were used to calculate the average hydrodynamic radii (*R*_h_) distributions via the Stokes-Einstein-Equation.

### Small-angle X-ray scattering (SAXS)

SAXS data were collected at the EMBL beamline P12 at PETRA III (DESY, Hamburg, Germany)^50^. A 2D photon-counting Pilatus 2 M pixel detector (Dectris) with a momentum transfer range 0.03 nm^−1^ < s < 4.8 nm^−1^ ($s=4\pi \frac{sin\theta }{\lambda }$, where 2θ is the scattering angle) was applied. Data collection parameters are listed in Supplementary Table S2. The protein concentrations ranged from 1.4 to 7.7 mg ml^−1^ in 20 mM Tris pH 7.4, 300 mM NaCl, 1 mM DTT. Protein sample solutions and the corresponding buffer solutions were exposed to the X-rays in alternating order for twenty consecutive exposure frames of 45 ms each. Scattering intensities of individual exposures were averaged, and the latter scattering amplitudes were subtracted from the protein solution scattering amplitudes applying the automated data processing pipeline SASFLOW^51^. The resulting curves were further processed by PRIMUSqt^52^. Due to no significant changes in *R*_g_, *I*(0) and *MW* with increasing protein concentrations, the subsequent analysis was carried out based on the highest measured concentration (3.1 mg ml^−1^ BnCYP18-4, 4.2 mg ml^−1^ BnCYP18-5, 7.1 mg ml^−1^ BnCYP19-1, 7.7 mg ml^−1^ AtCYP19-3). The *R*_g_ values and the maximum dimensions (*D*_max_) of native CYPs were obtained from AUTORG and the particle pair distance distribution functions *P*(*r*) were calculated via GNOM respectively^52^. DAMMIF was applied to generate 20 individual a*b initio* bead models for each protein sample^53^. The models are well superimposable as verified by their normalised spatial discrepancy (NSD). A bovine serum albumin reference solution (5 mg ml^−1^; 66.2 kDa) in 50 mM HEPES pH 7.5 was used to calibrate and verify the beamline operation.

### Crystallisation

Cyclosporin A (Sigma-Aldrich) was dissolved in pure DMSO and slowly added to the protein after tag cleavage at a molar ratio of 2:1 (CsA:protein). The CYP:CsA complex was mixed with an equal volume of reservoir solution. Initially, 800 different crystallization conditions were screened using the vapour diffusion and microbatch technique, respectively. X-ray suitable crystals of BnCYP19-1 in complex with CsA were obtained from sitting drop vapour diffusion crystallisation trials applying a protein concentration of 10 mg ml^−1^. The reservoir contained 2.4 M sodium malonate, pH 7.0. Bipyramidal crystals grew to a maximum size of approximately 200 × 200 × 200 µm^3^ within 3 weeks and were cryo-protected prior to X-ray data collection by slowly supplementing the drop with glycerol to a final concentration of 12% (v/v) on a micromesh.

### Diffraction data collection and processing

Diffraction data of BnCYP19-1 in complex with CsA were collected at the EMBL beamline P14 (PETRA III, DESY, Germany) applying an X-ray wavelength of 1.0332 Å and using one loop-mounted and flash-cooled crystal at 100 K. A single crystal was exposed to the beam at a rotation increment of 0.1°. Indexing was performed by IMOSFLM^54^. The space group was assigned to be I4_1_22. Unit cell and refinement parameters are summarized in Table 2.

**Table 2:** X-ray structure analysis: data processing and refinement statistics.

Beamline	P14, PETRA III, Hamburg, Germany
**Data collection**	
Wavelength (Å)	1.0332
Space group	I4_1_22
Cell dimensions (Å/°)	a = 86.58, b = 86.58, c = 119.52 α = 90, β = 90, γ = 90
Resolution range (Å)	70.12–1.98 (2.08–1.98)
Total no. of reflections	90890
No. of unique reflections	16197
Solvent content (%)	59.9
*R*_merge_	10.1 (46.6)
Completeness (%)	99.9 (98.3)
Redundancy	5.6 (5.9)
*I*/*σ*(*I*)	9.2 (3.5)
CC_1/2_	99.7 (89.3)
**Refinement**	
*R* (%)	19.8
*R*_free_ (%)	23.5
No. of protein atoms	1279
No. of ligands	3
No. of ligand atoms	93
No. of water molecules	50
B-factor protein	32.83
B-factor CsA/malonate/ion	32.23/52.92/33.73
B-factor water	31.08
R.m.s.d. bond length	0.018
R.m.s.d. bond angle	2.011
**Ramachandran plot**	
Favoured region	95.8%
Allowed region	4.2%
Outliers	0%

Numbers in parenthesis refer to the outer resolution shell. Diffraction data of one single crystal was used for data processing.

### Structure refinement

Diffraction data obtained were reduced and scaled applying SCALA, part of the CCP4 suite^55^, and converted via FREEFLAG^56^. The phase problem was solved by molecular replacement using MOLREP^57^ and coordinates of pdb entry 4JJM. For iterative refinement the program REFMAC^58^ was applied and manual refinement was performed using COOT^59^. The quality of refinement and coordinates was verified using PROCHECK^60^ and the REDO server^61^.

### Homology modelling

The tertiary structure of *B*. *napus* and *A*. *thaliana* CYPs in the absence of an active site ligand based on the obtained high-resolution structure of BnCYP19-1 was modelled using SWISS MODEL^62,63^. For comparison I-TASSER^64^ was applied and these models were also fitted into *ab initio* shapes.

### *In silico* ligand docking

The Flexidock™ subprogram implemented in Sybyl-X 1.2 (Tripos International) was applied for docking studies in order to determine the epitope of a substrate peptide at the active site of BnCYP19-1. In preparation, hydrogen atoms were added to the protein and Gasteiger-Hückel charges were calculated and assigned. The free energy of the protein was minimised over 500 cycles and 50 cycles for preparation of the ligand. The dielectric constant of the Tripos force field was set to 20.

## Supplementary information

Supplementary file

## Data Availability

The resolved macromolecular structure of BnCYP19-1 is deposited in the protein data bank via pdb ID 6HMZ.
