# Supplementary material for: Enzyme activity and structural features of three single-domain phloem cyclophilins from Brassica napus
Source: Sci Rep. 2019 Jun 27;9:9368. doi: 10.1038/s41598-019-45856-y (PMC6597583; doi:10.1038/s41598-019-45856-y)
Supplement: Supplementary file 1 — Supplementary file [file 41598_2019_45856_MOESM1_ESM.docx]

# Supplementary data

**Article title:** Enzyme activity and structural features of three single-domain phloem cyclophilins from Brassica napus

Authors

Patrizia Hanhart^a1^, Sven Falke^b1^, Marcel Garbe^a^, Victoria Rose^a^, Melanie Thieß^a^, Christian Betzel^b^, Julia Kehr^a*^

**^a^** Molecular Plant Genetics, Universität Hamburg, Institute of Plant Science and Microbiology, Ohnhorststraße 18, 22609, Hamburg, Germany

**^b^** Laboratory for Structural Biology of Infection and Inflammation, Institute of Biochemistry and Molecular Biology, Universität Hamburg, c/o DESY, Notkestraße 85, 22603 Hamburg, Germany

*^1^authors contributed equally*

***Corresponding author(s) details:** Prof. Dr. Julia Kehr, Molecular Plant Genetics, Universität Hamburg, Institute of Plant Science and Microbiology, Ohnhorststraße 18, 22609, Hamburg, Germany

**Corresponding author email:** julia.kehr@uni-hamburg.de

Supplementary **Table S1:** Primers for gene amplification and cloning.

| **Primer name** | **Sequence (5'-3')** |
| --- | --- |
| AtCYP18-3_for | GATCCATATGGCGTTCCCTAAGGTATAC |
| AtCYP18-3_rev | GATCCTCGAGCTAAGAGAGCTGACCACAATC |
| AtCYP19-3_for | GATCCATATGGCGAATCCTAAAGTCTTC |
| AtCYP19-3_rev | GATCCTCGAGTTATGAACTTGGGTTCTTGAGC |
| BnCYP18-4_for | GATCCATATGGCGTTTCCTAAAGTTTTC |
| BnCYP18-4_rev | GATCCTCGAGCTAAGAGAGCTGACCAC |
| BnCYP19-1_for | GATCCATATGGCAAACCCGAAGGTC |
| BnCYP19-1_rev | GATCCTCGAGCTATTCCTTGTTTGAAACTTCTG |

Supplementary **Table S2**: SAXS data collection and processing software.

| **Data collection parameters** | |
| --- | --- |
| Instrument | EMBL beamline P12^1^ |
| Wavelength (nm) | 0.124 |
| Sample to detector distance (m) | 3.1 |
| *s*-Range (nm^−1^) | 0.03–4.8 |
| Exposure time (s) | 0.05 × 20 |
| Concentration range (mg ml^−1^) | 1.8-7.7 |
| Temperature (K) | 283 |
| **Software employed** | |
| Primary data reduction and processing | SASFLOW pipeline^2^ |
| Basic analysis: Guinier plot, *P*(*r*) function | PRIMUSqt^3^ |
| *Ab initio* modelling | DAMMIF^4^ |
| Further data evaluation | CRYSOL^5^ |

**Supplementary Table S3**: List of hydrogen bonds and ionic interactions between BnCYP19-1 and its ligands.

| BnCYP19-1 residue/atom | Ligand name, residue/atom | Distance (Å) |
| --- | --- | --- |
| Arg 62/NH1 | CsA, Mle10/O | 3.1 |
| Arg 62/NH2 | CsA, Mle10/O | 3.0 |
| Gln70/NE2 | CsA, Bmt1/O | 3.0 |
| Asn109/O | CsA, Aba2/N | 3.1 |
| Ser110/OG | CsA, Aba2/O | 2.9 |
| Trp128/NE1 | CsA, Mle9/O | 2.9 |
| Leu53/O | Mg^2+^ | 2.6 |


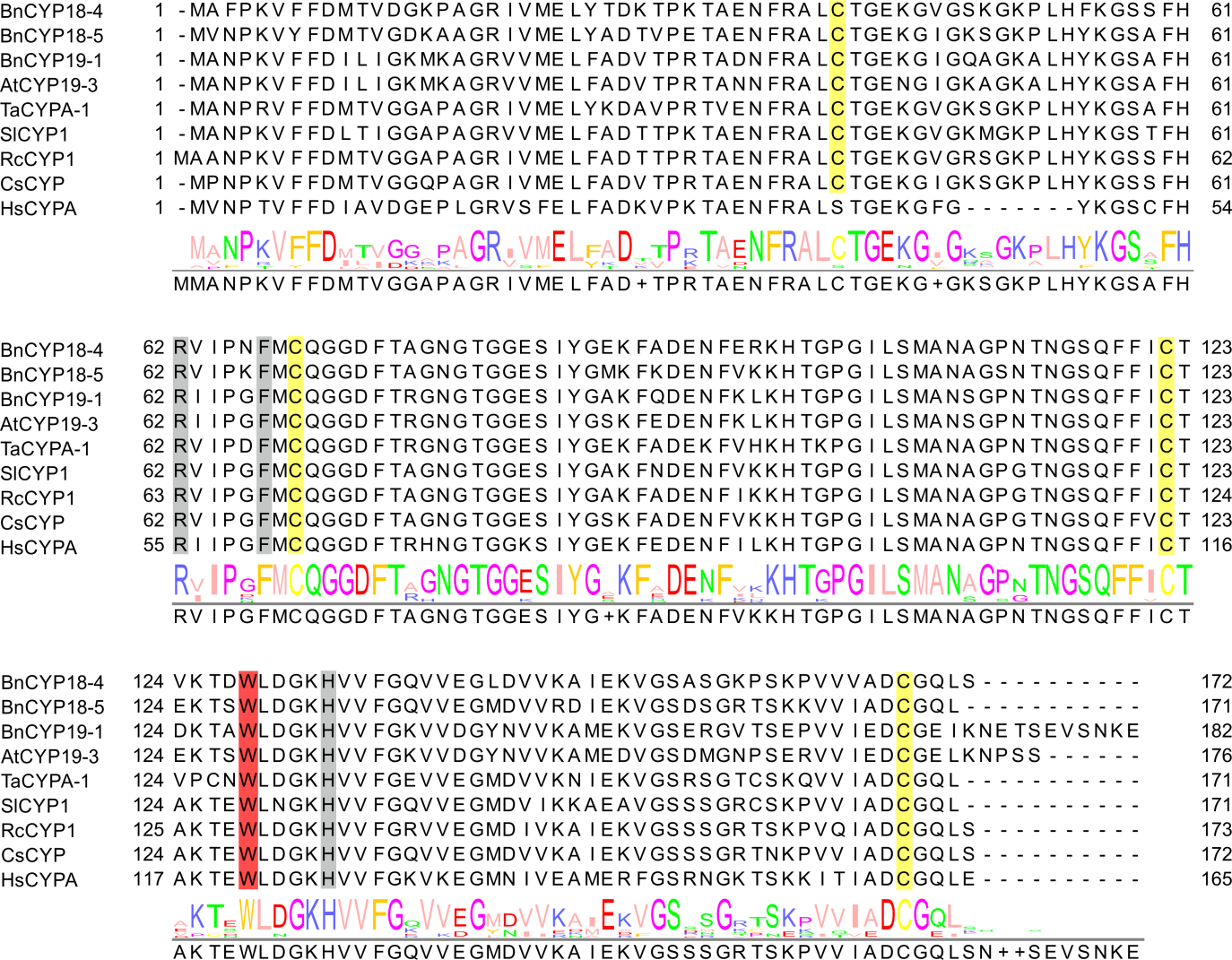


**Supplementary Figure S1**: Sequence comparison of single domain plant CYPs with HsCYPA. Single residues experimentally studied and reported to be associated with PPIase activity (HsCYPA Arg55, Phe60, His126^6^) are marked in grey and with CsA binding (HsCYPA Trp121^6^) in red. Four cysteine residues are conserved among plant CYPs (yellow) and two of them are reported to form a disulphide bond (CsCYP Cys40 and Cys168^7^).


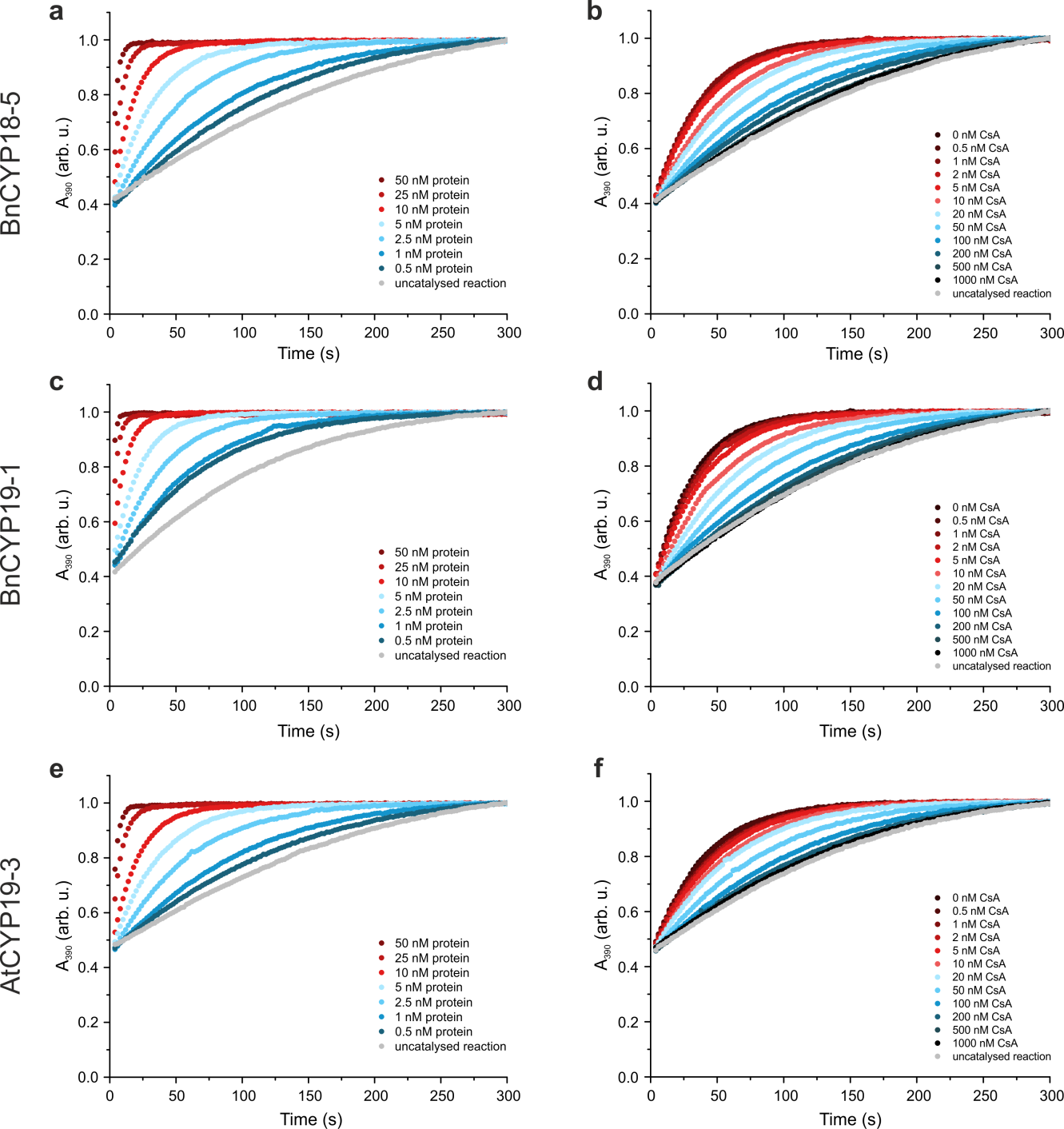


**Supplementary Figure S2**: PPIase assay normalised raw data of BnCYP18‑5, BnCYP19‑1, AtCYP19‑3 from activity (a, c, e) and inhibition (b, d, f) experiments.


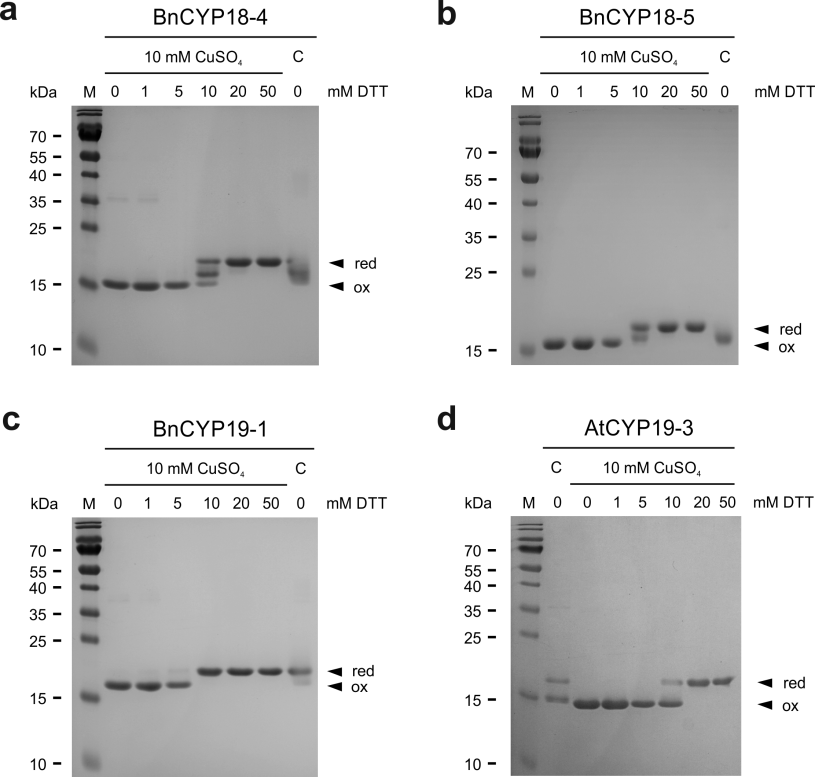


**Supplementary Figure S3**: Full size gels showing the redox-dependent migration pattern of (a) BnCYP18‑4, (b) BnCYP18‑5, (c) BnCYP19‑1, and (d) AtCYP19‑3. C = untreated control.


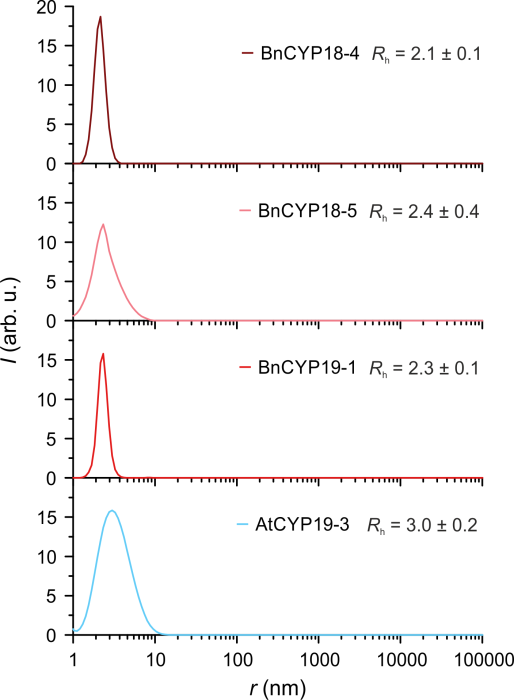


**Supplementary Figure S4**: DLS size distribution profiles. All investigated proteins were subjected to DLS prior to SAXS. The size distributions show the monodispersity and purity of all four protein samples.


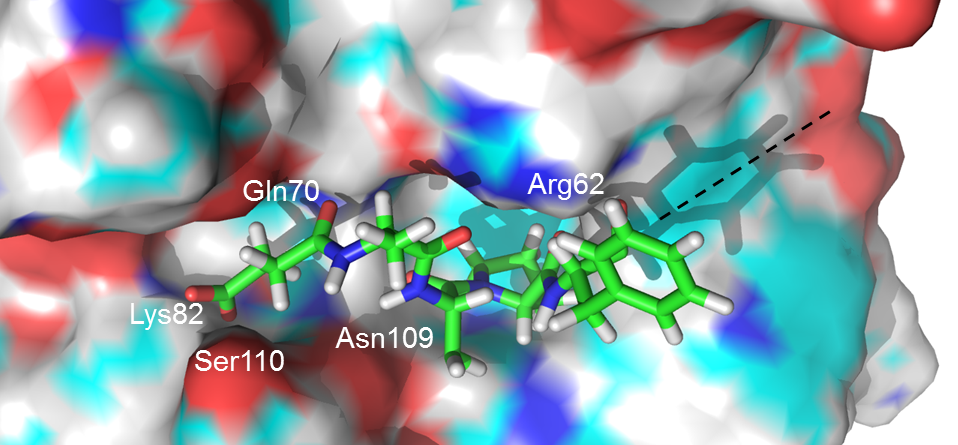


**Supplementary Figure S5**: Active site of BnCYP19-1 occupied by Suc-AAPF as investigated *in silico*. Amino acids involved in putative hydrogen bonds are labelled. The phenylalanine of the substrate peptide is forming a hydrogen bond via its backbone carbonyl oxygen independent of the phenyl side chain. The extended shape of the binding cleft strongly suggests the capability to recognise and bind longer substrates (dashed line).


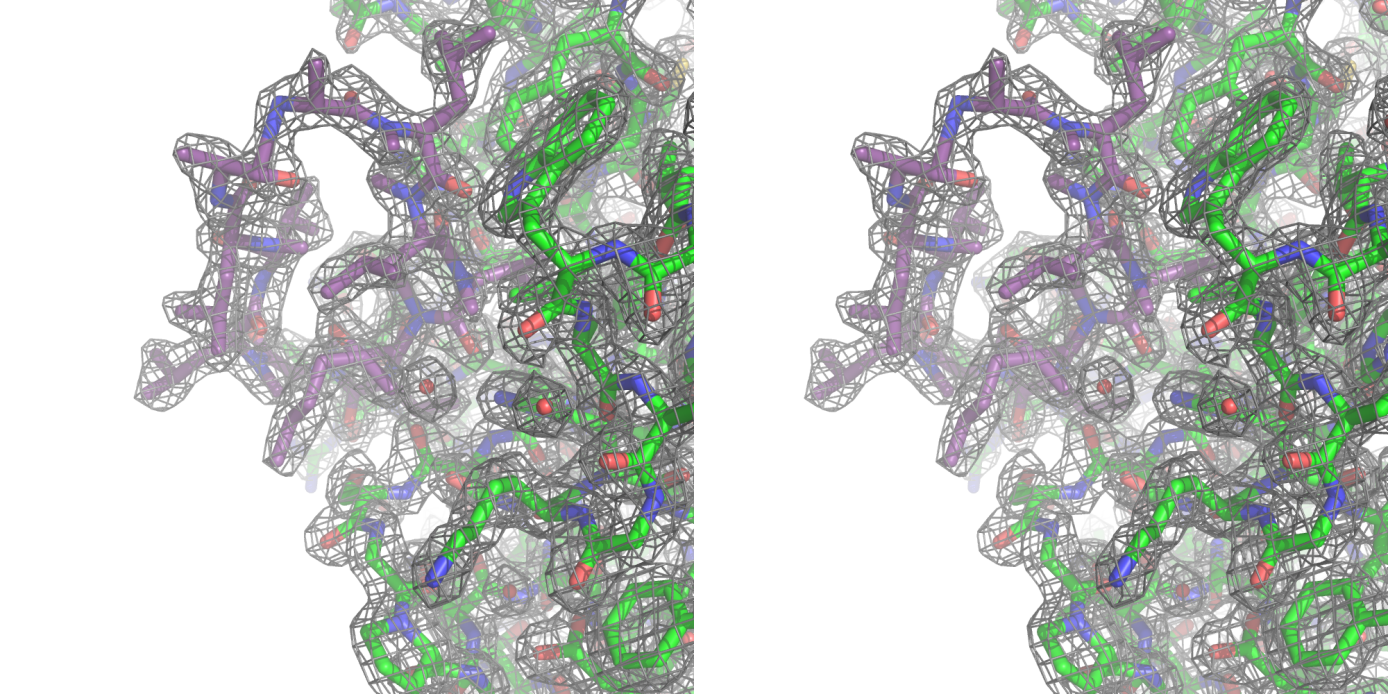


**Supplementary Figure S6 (cross-eye stereo):** Experimental 2Fo-Fc electron density map at 1.98 Å resolution showing a part of BnCYP19-1 binding to CsA (carbon atoms in purple). The stick representation of the protein is showing carbon atoms in green, nitrogen in blue and oxygen in red. The quality of these data ensured the correct assignment and positioning of the ligand with high confidence for further interpretation.

## References

1 Blanchet, C. E. *et al.* Versatile sample environments and automation for biological solution X-ray scattering experiments at the P12 beamline (PETRA III, DESY). *J. Appl. Crystallogr.* **48**, 431-443, doi:10.1107/S160057671500254X (2015).

2 Franke, D., Kikhney, A. G. & Svergun, D. I. Automated acquisition and analysis of small angle X-ray scattering data. *Nucl. Instrum. Methods Phys. Res.* **689**, 52-59, doi:10.1016/j.nima.2012.06.008 (2012).

3 Franke, D. *et al.* ATSAS 2.8: a comprehensive data analysis suite for small-angle scattering from macromolecular solutions. *J. Appl. Crystallogr.* **50**, 1212-1225, doi:10.1107/S1600576717007786 (2017).

4 Franke, D. & Svergun, D. I. DAMMIF, a program for rapid ab-initio shape determination in small-angle scattering. *J. Appl. Crystallogr.* **42**, 342-346, doi:10.1107/S0021889809000338 (2009).

5 Svergun, D. I., Barberato, C. & Koch, M. H. J. CRYSOL - a program to evaluate x-ray solution scattering of biological macromolecules from atomic coordinates. *J. Appl. Crystallogr.* **28**, 768-773, doi:10.1107/S0021889895007047 (1995).

6 Zydowsky, L. D. *et al.* Active site mutants of human cyclophilin A separate peptidyl-prolyl isomerase activity from cyclosporin A binding and calcineurin inhibition. *Protein Sci.* **1**, 1092-1099, doi:10.1002/pro.5560010903 (1992).

7 Campos, B. M. *et al.* A redox 2-Cys mechanism regulates the catalytic activity of divergent cyclophilins. *Plant. Physiol.* **162**, 1311-1323, doi:10.1104/pp.113.218339 (2013).
